# Antifungal activity of 8-methoxynaphthalen-1-ol isolated from the endophytic fungus *Diatrype palmicola* MFLUCC 17-0313 against the plant pathogenic fungus *Athelia rolfsii* on tomatoes

**DOI:** 10.7717/peerj.9103

**Published:** 2020-05-04

**Authors:** Chutima Tanapichatsakul, Acharavadee Pansanit, Sakon Monggoot, Siraprapa Brooks, Surasak Prachya, Prasat Kittakoop, Parinya Panuwet, Patcharee Pripdeevech

**Affiliations:** 1 School of Science, Mae Fah Luang University, Muang, Chiang Rai, Thailand; 2 Institute of Beauty and Health Sciences Co.,Ltd. (IBHS/BKK), Sakdibhornssup Bldg., Makkasan, Ratchathewi, Bangkok, Thailand; 3 Chulabhorn Research Institute, Laksi, Bangkok, Thailand; 4 Program in Chemical Sciences, Chulabhorn Royal Academy, Chulabhorn Graduate Institute, Laksi, Bangkok, Thailand; 5 Center of Excellence on Environmental Health and Toxicology (EHT), CHE, Ministry of Education, Bangkok, Thailand; 6 Laboratory of Exposure Assessment and Development for Environmental Research, Department of Environmental Health, Rollins School of Public Health, Emory University, Atlanta, GA, United States of America; 7 Center of Chemical Innovation for Sustainability (CIS), Mae Fah Luang University, Muang, Chiang Rai, Thailand

**Keywords:** Antifungal activity, 8-methoxynaphthalen-1-ol, Endophytic fungi, Plant pathogen, Tomato, Southern blight, Diatrype, *Athelia rolfsii*

## Abstract

Thirty-four endophytic fungal isolates were obtained from the leaves of the medicinal plant *Polyscias fruticosa*, and their antagonistic activities against the growth of the common tomatoes plant pathogenic fungus* Athelia rolfsii* were initially screened using a dual culture assay. The endophytic fungus MFLUCC 17-0313, which was later molecularly identified as *Diatrype palmicola*, displayed the highest inhibition percentage (49.98%) in comparison to the others. This fungus was then chosen for further evaluation. Its culture broth and mycelia from a 10 L scale were separated and extracted using ethyl acetate, methanol, and hexane. Each extract was tested for antifungal activity against the same pathogen using a disc diffusion assay. Only the crude hexane extract of fungal mycelium showed antifungal activity. The hexane extract was fractioned using sephadex gel filtration chromatography and each fraction was tested for antifungal activity until the one with the highest inhibition percentage was obtained. The bioactive compound was identified as 8-methoxynaphthalen-1-ol using nuclear magnetic resonance spectroscopy and mass spectrometry. The minimum inhibition concentration of 8-methoxynaphthalen-1-ol was demonstrated at 250 µg/mL against the selected pathogen. Using the leaf assay, the solution of 8-methoxynapthalen-1-ol was tested for phytotoxic activity against *A. rolfsii* and was found to have no phytotoxic effects. These results showed that 8-methoxynaphthalen-1-ol has the potential for controlling the growth of *A. rolfsii*, the cause of Southern blight disease on tomatoes. This study may provide the foundation for future use of this compound as a biofungicide.

## Introduction

Tomato (*Solanum lycopersicum* L.) is among the most popular and extensively consumed economic vegetable crop in the world. Each year, more than 100 million MT of tomatoes are produced by the major producers: China, USA, India, and Turkey (Singh, Singh & Kumar, 2017). Production of tomatoes requires a delicate system. The quantity and quality of tomatoes are affected by various diseases across the pre- and post-harvest periods (Singh, Singh & Kumar, 2017). Presently, there are more than 200 known diseases reported during the production of tomatoes, resulting in varying losses in overall product quantity and market values (Nowicki, Kozik & Foolad, 2013).

One of the most destructive tomato diseases is Southern blight, which is caused by infection with the basidiomycete fungus *A. rolfsii* (syn. *Sclerotium rolfsii*) and typically results in significant economic loss (Banyal, Mankotia & Sugha, 2008). This disease is widespread in all tomato-growing areas throughout the world and appears at any stage of plant development during all seasons. The *A. rolfsii* fungus infects tomatoes directly and indirectly via wounds caused by nematodes or insects. Fruits, leaves, or branches that touch the soil may also be infected by the germinating sclerotia. Yield loss of tomato production caused by *A. rolfsii* infection has been reported to vary between 35% and 90% (Sikiroul et al., 2010).

Management of *A. rolfsii* infection has been performed using chemical fungicides such as benomyl, sancozeb, thiovit, mancozeb, and carbendazim (Yaqub & Shahzad, 2006). These fungicides are able to inhibit the mycelial growth of *A. rolfsii* (Yaqub & Shahzad, 2006; Dwivedi & Prasad, 2016). One of the most effective fungicides used for controlling *A. rolfsii* infection is mancozeb (Dwivedi & Prasad, 2016). However, use of mancozeb has resulted in residue contamination in soil and crops, which may impact the environment and the health of consumers (Paro et al., 2012; Kaye et al., 2015). Moreover, the metabolite or the degradation product of mancozeb, ethylenethiourea (ETU), is classified as possibly carcinogenic to humans (Group 2B) (IARC, 1987). Exposure to mancozeb and ETU is a serious health concern. Therefore, a new fungicide that can control the growth of *A. rolfsii*, is environmentally friendly without impact on the environment and human health is highly needed.

Microorganisms are capable of producing bioactive compounds that display antimicrobial activities. A large number of microorganisms, especially endophytic fungi, have been studied for the production of their metabolic products that can be used as biocontrol agents to replace the use of synthetic fungicides (Gouda et al., 2016). For instance, (3R,4R,6R,7S)-7-hydroxyl3,7-dimethyl-oxabicyclo[3.3.1]nonan-2-one and (3R,4R)-3-(7-methylcyclohexenyl)-propanoic acid were isolated from the endophytic fungus *Pestalotiopsis foedan* and showed significant antifungal activities against *Botrytis cinerea* and *Phytophthora nicotianae*. In addition, cladosporin, isolated from the endophytic fungus *Cladosporium cladosporioides*, was shown to inhibit the growth of *Colletotrichum acutatum*, *C. fragariae*, *C. gloeosporioides*, and *Phomopsis viticola* (Deshmukh et al., 2018).

*Polyscias fruticosa* (L.) Harms belongs to the family Araliaceae and is widely used in many countries of Southeastern Asia for medicinal and food purposes (Vo et al., 1998). This plant was reported to contain bioactive compounds such as saponins, oleanolic acid, and polyacetylenes that have antibacterial, antifungal, anti-inflammatory, antitoxin, antipyretic, molluscicidal, and anti-diabetic activities (Hanh, Dang & Dat, 2016; Boye et al., 2018). As such for the first time, the endophytic fungi that colonize within this plant were isolated and tested for their antifungal activities.

Because endophytic fungi have been important resources for antifungal compounds against phytopathogens, the present study aimed to isolate endophytes from the leaves of *Polyscias fruticosa*, screen for their antimicrobial activity against *A. rolfsii*, and isolate the bioactive compound(s) from the crude extract(s) that showed significant antimicrobial activity. The obtained antifungal compound may be used as an alternative agent to control infection of *A. rolfsii* on tomatoes.

## Materials & Methods

### Plant material

Healthy leaves of *P. fruticosa* were collected in January 2017 from Mae Chan District, Chiang Rai Province, Thailand (99°70′56″E, 20°10′36″N) for endophytic fungi isolation. The botanical specimens of this plant (MFL10011) were deposited at the Mae Fah Luang Botanical Garden of Mae Fah Luang University, Thailand.

### Isolation of endophytic fungi

The isolation procedures were performed following the method described by Atiphasaworn et al. (2017). Healthy leaves of *P. fruticosa* were washed with running water for 2 min followed by 70% ethanol for 10 s and then sterilized with 1% sodium hypochlorite for 30 s and washed twice with sterile water. Sterilized leaf tissues were sectioned into 0.5 cm^2^ pieces. Approximately 100 pieces were randomly selected and evenly distributed between 30 Petri dishes containing potato dextrose agar (PDA) mixed with chloramphenicol (30 µg/mL) to prevent bacterial growth. Petri dishes were sealed with sterile Parafilm prior to incubation at room temperature (27 °C) for 5 days. Petri dishes were observed daily, and the individual hypha tips of the emerging fungal colonies were sub-cultured on fresh PDA plates at room temperature (27 °C). All isolates were deposited at the Center of Excellence in Fungal Research, Mae Fah Luang University, Thailand (Voucher specimen no. MFLUCC 17-0280-MFLUCC 17-0313).

### Isolation and molecular identification of the phytopathogen *A. rolfsii*

For antifungal activity testing, the pathogen *A. rolfsii* was isolated from infected *S. lycopersicum* leaves following a slightly modified method described by Adesegun et al. (2013). The infected *S. lycopersicum* leaves were collected in July 2016 from a tomato farm in Sansai district, Chiang Mai province, Thailand (98°95′58″E, 19°10′13″N). The infected leaves were washed with sterile distilled water and cut into five mm^2^ segments. Their surface was sterilized using 1% sodium hypochlorite for 1 min and rinsed with sterilized distilled water three times. The segments were placed on Petri dishes to dry at room temperature (27 °C) and further placed on 10 Petri dishes containing PDA mixed with chloramphenicol (30 µg/mL) to prevent bacterial growth. The dishes were incubated at room temperature (27 °C) and observed for 5 days. The isolate was sub-cultured to obtain a pure culture of *A. rolfsii*, which was stored at room temperature. The pathogen was identified based on its morphological characteristics (Adesegun et al., 2013). The pathogenicity of this *A. rolfsii* isolate was confirmed by Koch’s postulates. The aerial mycelium of *A. rolfsii* isolate was scraped from the PDA surface and pulverized with a mortar and pestle to obtain a mycelium pulp. The genomic DNA was extracted following the cetyltrimethyl ammonium bromide method (Elias et al., 2018). Two universal primers, ITS4 (5′-TCCTCCGCTTATTGATATGC-3′) and ITS5 (5′-GGAAGTAAAAGTCGTAACAAGG-3′), were used. Polymerase chain reactions (PCR) were performed at 95 °C for 5 min, followed by 40 cycles of 95 °C for 50 s, 52 °C for 50 s, 72 °C for 50 s, and 72 °C for 10 min on a PeqSTAR 2×  thermal cycler (Peqlab, Germany). The PCR-amplified product was analyzed by gel electrophoresis on 1% agarose gels stained with ethidium bromide under UV light and purified using NucleoSpin^®^ Gel and PCR Clean-up Kit (Macherey-Nagel, Germany). DNA sequencing was performed on an automated sequencing system ^1^ST Base (Malaysia). The obtained sequence was used as a query to search for sequences in GenBank using the BLAST program (Basic Local Alignment Search Tool, https://blast.ncbi.nlm.nih.gov/Blast.cgi).

### In vitro antifungal screening by dual culture assay

Endophytic fungal isolates were screened for their antifungal activity against the growth of *A. rolfsii* using the *in vitro* dual culture assay. PDA was used as a medium. For each combination of the pathogen and endophytic fungus, a mycelial plug (five mm in diameter) of *A. rolfsii* and fungal isolate were placed on Petri dishes containing PDA. The plates were incubated at 30 °C. Petri dishes inoculated only with *A. rolfsii* were used as negative controls. All plates were incubated for seven days until the negative control plates were completely covered with *A. rolfsii* mycelia The growth inhibition of each endophytic fungus against the tested pathogen was expressed as the inhibition percentage of a radical growth relative to the controls, using the following formula: Inhibition percentage (%) = 100 × [1 −(growth area of the treatment/growth area of the control)]. All experiments were conducted in triplicate.

### Fungal cultivation and extraction

From the results of the dual culture assay, the endophytic fungus MFLUCC 17-0313, which showed the strongest *in vitro* antifungal activity against *A. rolfsii*, was selected for further cultivation, extraction, isolation, and characterization of the active compound. This isolate was cultured in potato dextrose broth (PDB) using a method described by Atiphasaworn et al. (2017). Five mycelial agar plugs ( five mm diameter, seven days old) of the endophytic fungus MFLUCC 17-0313 were placed in each 1,000 mL Erlenmeyer flask (40 flasks total) containing 250 mL of PDB. The flasks were incubated at room temperature (27 °C) for 28 days without shaking. The culture broth was separated from fungal mycelia by filtration through filter paper. The culture broth was extracted with an equal volume of ethyl acetate three times. The extracts were concentrated under vacuum using a rotary evaporator to produce dried broth extract. The mycelial fraction was macerated with methanol for two days and further concentrated using a rotary evaporator. They were dissolved in 200 mL of distilled water before being partitioned with an equal volume of hexane and ethyl acetate three times, respectively. The obtained hexane and ethyl acetate extracts were collected, and combined. Residue water was eliminated using anhydrous sodium sulfate. The extracts were evaporated using a rotary evaporator to obtain dried extracts from the hexane and ethyl acetate fraction, respectively. In addition, the same mycelia was macerated in dichloromethane for 2 days. The solution was filtered to obtain the dichloromethane extract. Residue water was eliminated using anhydrous sodium sulfate. The extract was evaporated using a rotary evaporator to obtain the dried dichloromethane extract. All extracts were stored at 4 °C until use.

### In vitro antifungal activity by disc diffusion assay

The antifungal activity of each crude extract obtained from the endophytic fungus MFLUCC 17-0313 was screened against *A. rolfsii* using the disc diffusion assay, as described by Tanapichatsakul et al. (2019). The dried crude extracts were dissolved using the individual extraction solvent to yield a concentration of 5 mg/mL. A plug of pathogenic fungal culture (five mm diameter, 5 days old) was placed on the center of the sterilized PDA plates and incubated at 30 °C for 2 days. A 6-mm filter paper disc (no. 3; Whatman, Maidstone, UK) moistened with 30 µL of crude extract solution was placed onto the surface of culture plates. Each crude extract solution was tested individually. The plates were incubated at 30 °C for 7 days and observed daily. Ethyl acetate, hexane, and dichloromethane were used as negative controls, while an amphotericin B solution (dissolved in distilled water to yield a concentration of 5 mg/mL) was used as a positive control (Sigma-Aldrich, USA). The growth inhibition of all endophytic fungal extracts against the tested fungal pathogen was expressed as the inhibition percentage of a radical growth relative to the control, using the following formula: inhibition percentage (%) = 100 × [1 −(radical growth of the treatment (mm)/radical growth of the control (mm))]. All experiments were conducted in triplicate.

### Isolation of antifungal compound from the endophytic fungus MFLUCC 17-0313

According to the results of the disc diffusion assay, only the crude hexane extract of fungal mycelia displayed antifungal activity against *A. rolfsii*. Therefore, it was fractioned using Sephadex LH-20 (GE Health Care Bio-Sciences AB, USA) gel filtration chromatography (3 × 84 cm column) and eluted with a mixture of methanol and dichloromethane (1:1 ratio) to yield a total of 24 fractions (F1–F24). All fractions were evaporated to dryness. The dried residue was dissolved with hexane to yield a solution with a concentration of 5 mg/mL. Each solution was tested for antifungal activity using the disc diffusion method. The F19 solution exhibited the strongest antifungal activity. Thin layer chromatography (TLC) was used to determine the purity of this solution. The TLC result indicated that the F19 solution contains only one compound. Therefore, the F19 fraction was subject to structural identification using ^1^H and ^13^C nuclear magnetic resonance (NMR) spectroscopy. ^1^H- and ^13^C-NMR spectra were recorded at 300 and 75 MHz, respectively, using a Bruker AVANCE 300 spectrometer with CDCl_3_ as an internal standard. The obtained NMR data was compared to those found in the report of Emre (2018). A mass spectrum of the isolated compound was obtained using a Bruker MicroTO*F*_LC_ mass spectrometer (Bruker, United Kingdom).

### Determination of the minimum inhibitory concentration

The minimum inhibitory concentration (MIC) value of the isolated antifungal compound against *A. rolfsii* was determined. Serial dilution was used to prepare seven concentrations (1,000, 500, 250, 125, 62.5, and 31.25 µg/mL) of the active compound in hexane. MIC was defined as the lowest concentration of the active compound that displayed visible growth inhibition of the pathogen on the agar plate. All experiments were performed in triplicate and the results were compared with the amphotericin B solution prepared at a concentration of 1,000 µg/mL.

### Phytotoxic assay

The phytotoxicity of the active compound was assayed using a leaf disk and absorption assay according to a method by Reveglia et al. (2019) with modifications.

The leaf disk assays were performed using fourteen-day old *S. lycopersicum* leaves. Disks of the lamina of plant leaves (six mm diameter) were prepared using a sharp corkborer. The compound was dissolved in a mixture of 0.25% tween 20 with 4% methanol into final concentrations of 1,000, 500, 250, and 125 µg/mL. The leaf disks were immersed in 1.0 mL of different compound solutions. After 18 h, the disks were washed with distilled water. The symptoms were visually observed using a 0 to four scale, where a 0 was no effect, 1 was area of necrotic spots <50%, 2 was area of necrotic spots 50–70%, 3 was area of necrotic spots 70–90%, and 4 was area of necrotic spots >90%. Distilled water and a mixture of 0.25% tween 20 with 4% methanol were used as negative controls whereas glyphosate dissolved in distilled water was used as a positive control. The experiment was carried out in triplicate.

In the leaf absorption assays, 14-day old *S. lycopersicum* leaves were cut and then placed in a tube containing 1.0 mL of compound solutions. The compound solutions were prepared by dissolving in a mixture of 0.25% tween 20 with 4% methanol and tested at four different concentrations: 1,000, 500, 250, and 125 µg/mL. After 48 h, the leaves were moved to a new tube containing distilled water. The symptoms were visually observed after 48 h using a 0 to four scale, where a 0 was no effect, 1 was area of necrotic spots <50%, 2 was area of necrotic spots 50–70%, 3 was area of necrotic spots 70–90%, and 4 was area of necrotic spots >90%. Distilled water and a mixture of 0.25% tween 20 with 4% methanol were used as negative control whereas glyphosate dissolved in distilled water were used as positive control. The experiment was carried out in triplicate.

### Identification of the endophytic fungus MFLUCC 17-0313

The molecular identification of endophytic fungus MFLUCC 17-0313 was preliminarily performed using the method as described in section of molecular identification of the phytopathogen *A. rolfsii*. The sequences of representative taxa in Diatrypaceae used in our phylogenetic analyses were selected from GenBank based on the BLASTn searches and recently published data (Senwanna et al., 2017; Konta et al., 2020). ITS and β-tubulin sequence datasets were selected to construct the phylogenetic tree. The combined ITS and β-tubulin sequence data were initially aligned by using MAFFT version 7 (Katoh, Rozewicki & Yamada, 2017; http://mafft.cbrc.jp/alignment/server/). The alignment was checked and improved in BioEdit v. 7.0.5.3 (Hall, 1999). The final alignment of the combined ITS and β-tubulin sequence datasets was analyzed and inferred the phylogenetic tree based on maximum likelihood (ML) and Bayesian inference analyses (BI). The estimated evolutionary model of Bayesian inference and maximum likelihood were performed independently for each locus using MrModeltest v. 2.3 (Nylander, 2004). The best-fit model is resulted as GTR+I+G model for each locus under the Akaike Information Criterion (AIC). Maximum likelihood (ML) analyses was performed by using the RAxML-HPC2 on XSEDE (v. 8.2.12) (Stamatakis, 2014) via the CIPRES Science Gateway platform (Miller, Pfeiffer & Schwartz, 2010). Bayesian inference (BI) analysis was performed by MrBayes on XSEDE, MrBayes 3.2.6 (Huelsenbeck & Ronquist, 2001) via the CIPRES Science Gateway platform (Miller, Pfeiffer & Schwartz, 2010). Bayesian posterior probabilities (BYPP) (Rannala & Yang, 1996; Zhaxybayeva & Gogarten, 2002) were evaluated by Markov Chain Monte Carlo sampling (BMCMC). Two parallel runs were conducted using the default settings, but with the following adjustments: Six simultaneous Markov chains were set up at 5,000,000 generations. Trees were sampled every 100th generation. The first 10% of generated trees representing the burn-in phase were discarded and the remaining trees were used to calculate posterior probabilities of the majority rule consensus tree. The phylogenetic trees were figured in FigTree v.1.4.3 (Rambaut, 2016) and edited using Microsoft PowerPoint 2013 and converted to jpeg file in Adobe Photoshop CS6 version 13.0 (Adobe Systems. USA). The sequence of the fungus MFLUCC 17-0313 was also submitted to the NCBI GenBank (accession number MK329053).

### Statistical analysis

All experiments were performed in triplicate. Results were expressed as means with error bars for standard deviations. One-way analysis of variance (ANOVA), followed by a *t*-test, was used to determine the statistically significant difference in the experiments. A *P*-value of less than 0.05 was considered to indicate statistically significant differences.

## Results

### Isolation of endophytic fungi

Thirty-four fungal isolates were obtained from *P. fruticosa* leaves. Morphology of the endophytic fungi isolates are shown in Fig. S1.

### Screening of antifungal activity

**Figure 1:**
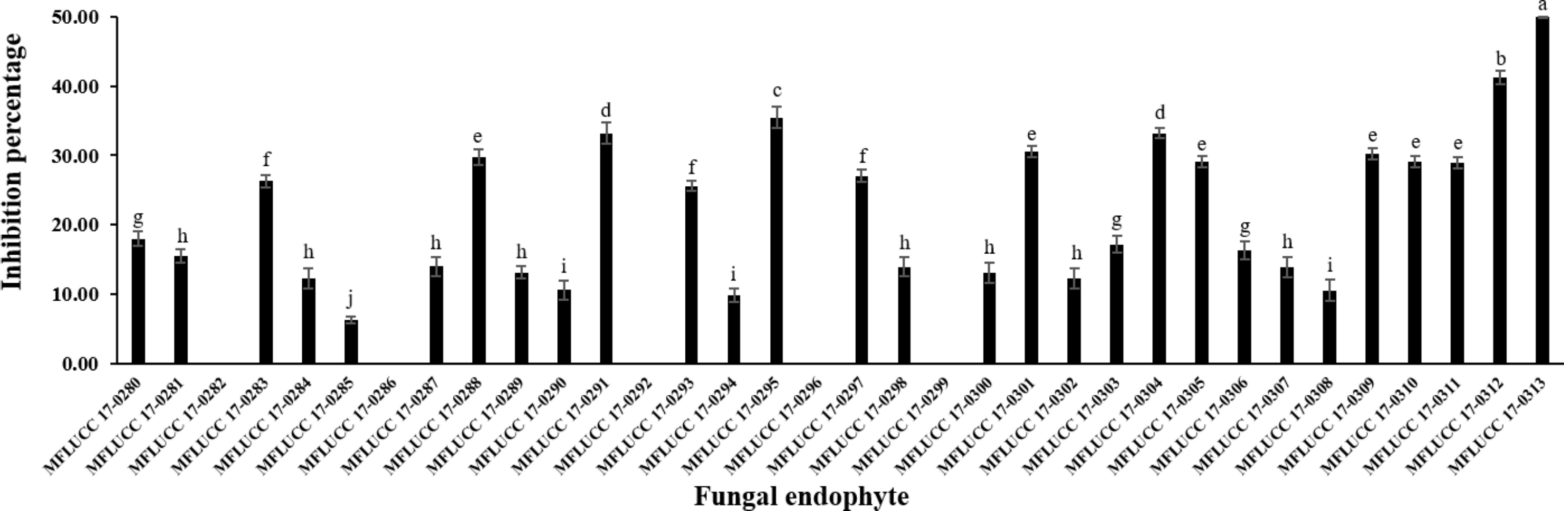
Inhibition percentage of the all endophytic fungi against *A. rolfsii* from dual culture assay. Each data indicates the average percentage of inhibition of endophytic fungus and a standard deviation value derived from a triplicate experiment. One-way analysis of variance (ANOVA), followed by a *t*-test, was used to determine the statistically significant difference.

**Figure 2:**
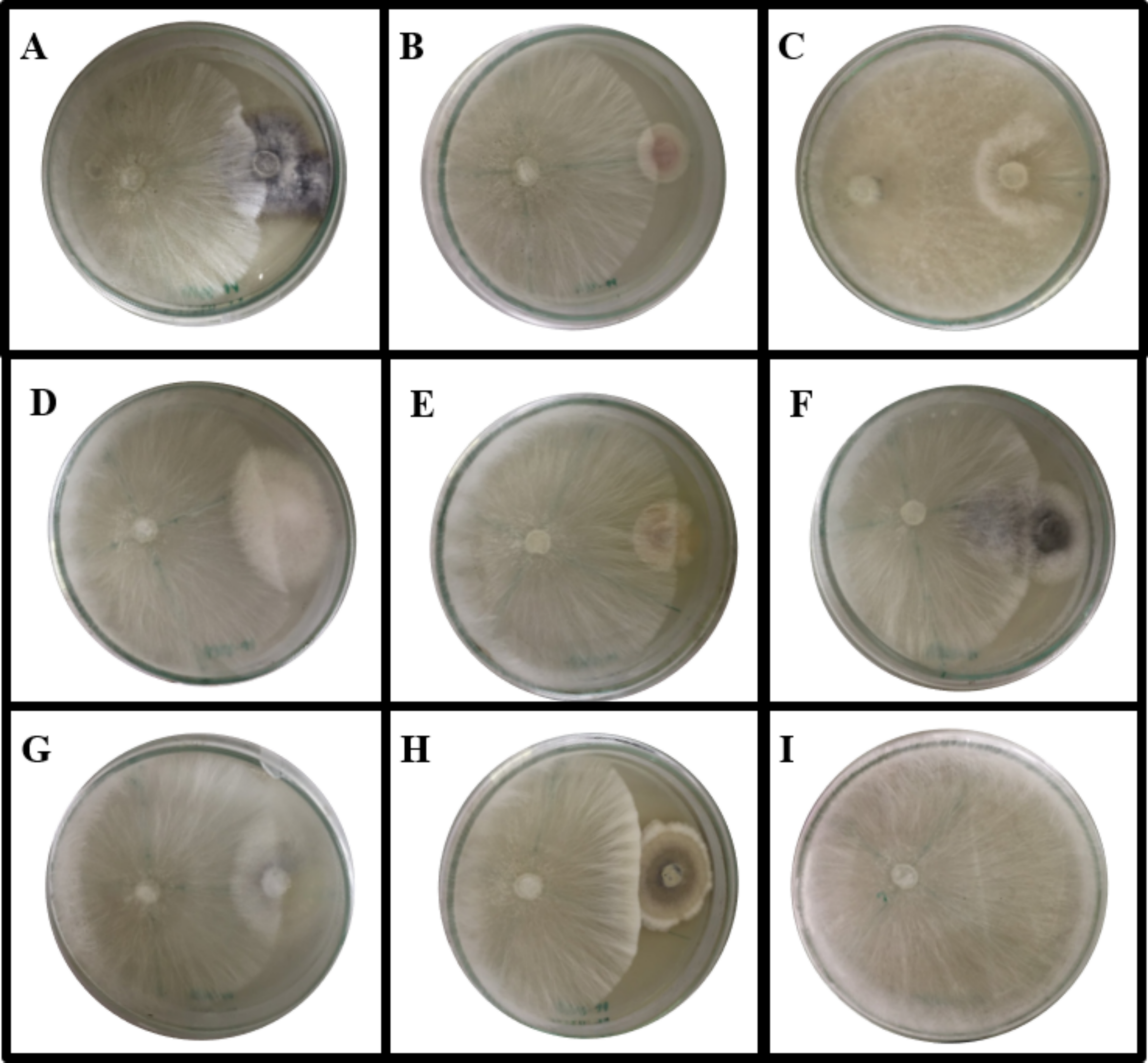
Dual culture plate assay between some endophytic fungi against the pathogen *A. rolfsii*. *A. rolfsii* was grown in PDA plates together with (A) MFLUCC 17-0281, (B) MFLUCC 17-0288, (C) MFLUCC 17-0296, (D) MFLUCC 17-0297, (E) MFLUCC 17-0298, (F) MFLUCC 17-0310, (G) MFLUCC 17-0312 and (H) MFLUCC 17-0313 while (I) represents *A. rolfsii* in PDA plate. The plates were cultivated for seven days at 30 °C.

The antifungal activity of each isolated endophytic fungi against the pathogen *A. rolfsii* was observed. Figure 1 and Table S1 show the inhibition percentage of each endophytic fungus against the pathogen *A. rolfsii* from dual culture assay. Antifungal activity of some endophytic fungi against *A. rolfsii* for seven days using dual culture assay is shown in Fig. 2. The percentage of growth inhibition ranged from 6.32% to 49.98%. The endophytic MFLUCC 17-0313 isolate possessed the highest inhibition percentage (49.98%). The mean inhibition percentage obtained from the endophytic fungus MFLUCC 17-0313 was significantly different from the other 33 isolates.

### Antifungal activity of crude extracts of the endophytic fungus MFLUCC 17-0313

The endophytic fungus MFLUCC 17-0313 was further cultured in PDB before its bioactive compound was extracted using different organic solvents. The crude extracts were tested for their antifungal activity against the pathogen *A. rolfsii*. Only the crude hexane extract of the fungal mycelium was found to exhibit a significant antifungal activity with an inhibition of 64.71%, while amphotericin B (prepared at 5 mg/mL) showed an inhibition percentage of 67.53% (Fig. S2). No antifungal activity against *A. rolfsii* was found in the other tested broth or mycelium extracts.

### Isolation and identification of the active compounds

**Figure 3:**
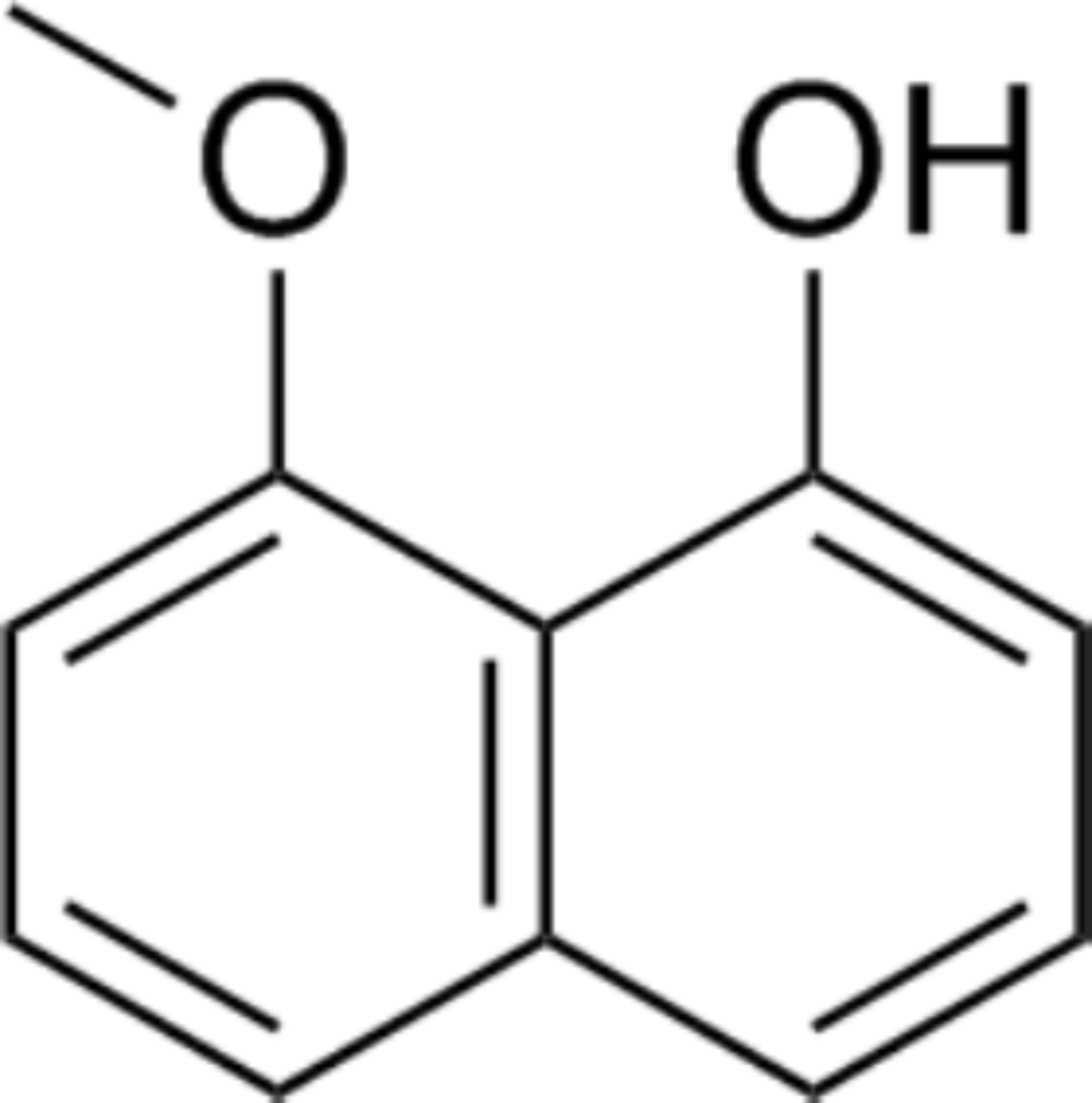
Chemical structure of 8-methoxynaphthalen-1-ol. This compound was isolated from *Diatrype palmicola* MFLUCC 17-0313 inhibited the plant pathogenic fungus *Sclerotium rolfsii* on tomatoes.

The crude hexane extract of fungal mycelium was fractioned to obtain the one containing the bioactive compound. Results from the disc diffusion method indicated that F19 fraction contains a bioactive compound with an inhibition percentage of 76.42 ± 0.04%. The results of antifungal activity of some fractions are shown in Fig. S3. The bioactive compound was concentrated and subjected to structural identification. The ^1^H NMR (400 MHz, CDCl_3_) results are as follows: δ 9.32 (1H, s), 7.42 (1H, dd, *J* = 0.5, 8.3 Hz), 7.35 (1H, t, *J* = 8.1 Hz), 7.33–7.28 (2H, m), 6.88 (1H, dd, *J* = 1.3, 7.4 Hz), 6.78 (1H, d, *J* = 7.6 Hz), and 4.06 (3H, s), while the ^13^C NMR (CDCl_3_, 100 MHz) results are also follows: δ 156.13, 154.45, 136.71, 127.70, 125.58, 121.84, 118.84, 115.04, 110.40, 103.86, and 56.08. These NMR data are in good agreement with those reported in Emre (2018). In addition, the mass spectrum of this compound showed an m/z of 175.0759, with a predicted chemical formula of C_11_H_11_O_2_. This compound was identified as 8-methoxynaphthalen-1-ol (Fig. 3). All NMR and mass spectral data are depicted in supplement information.

### MIC value

The MIC value of the isolated compound, 8-methoxynaphthalen-1-ol, was determined. The results revealed moderate antimicrobial activity of the compound against *A. rolfsii* with an MIC of 250 µg/mL. The result of antifungal activity of this compound is shown in Fig. S4.

### Phytotoxicity test

**Figure 4:**
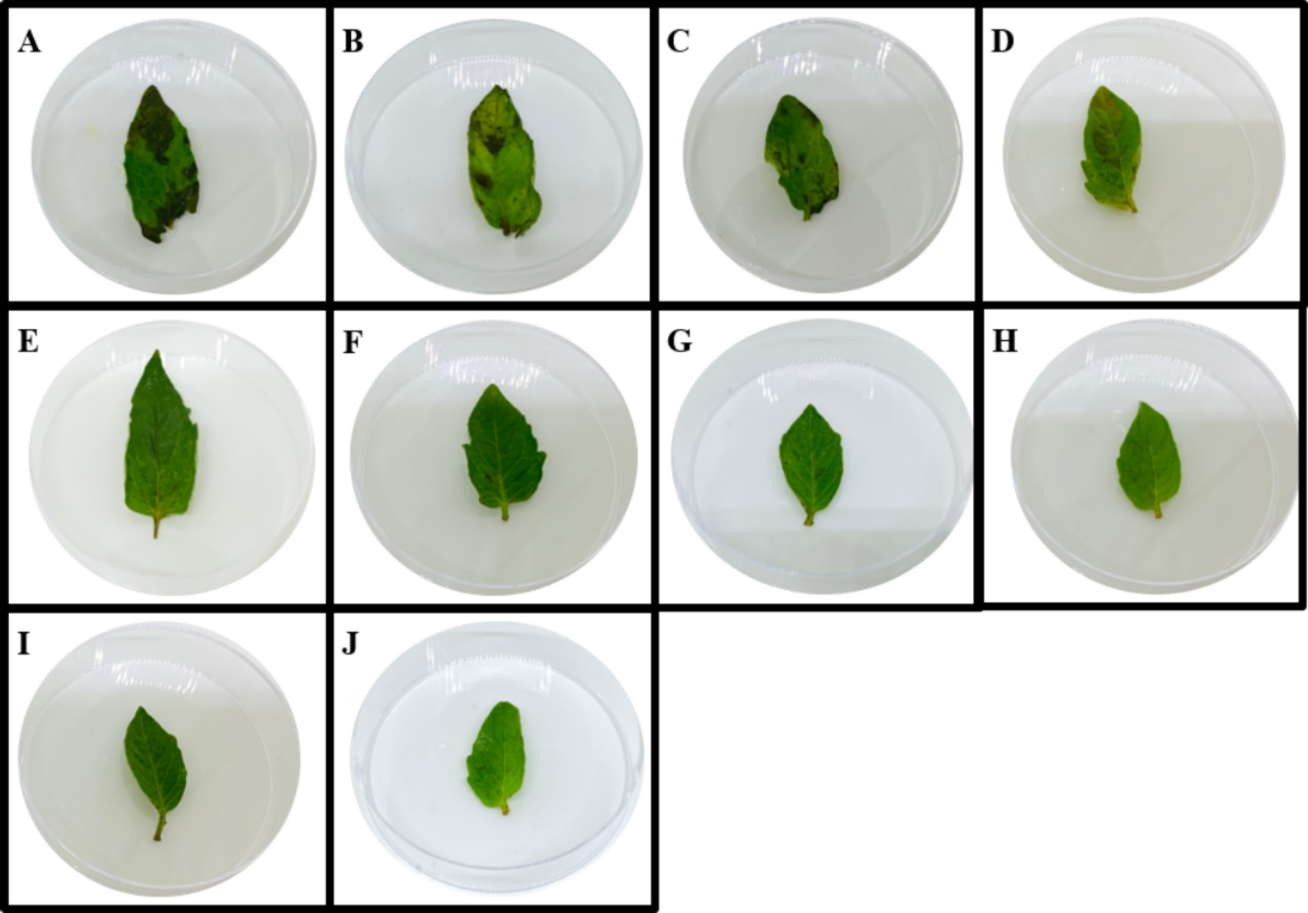
Symptoms caused by compounds toward *S. lycopersicum* leaf disks at different concentrations after 18 h. A-D represent symptoms caused by glyphosate at concentration of 1,000, 500, 250 and 125 µg/mL, E-H represent symptoms caused by 8-methoxy-napthlen-1-ol at concentration of 1,000, 500, 250 and 125 µg/mL, I represents symptoms caused by a mixture of 0.25% tween 20 with 4% methanol, J represents symptoms caused by distilled water.

In the present study, 8-methoxynaphthalen-1-ol, which showed a moderate antifungal activity against *A. rolfsii*, was further tested for its phytotoxic effect on plant leaves. Using the leaf disk and absorption assays, the development of symptoms after treatments with 8-methoxynaphthalen-1-ol at various concentrations were observed and compared to those obtained from glyphosate at the same concentrations. Distilled water and a mixture of 0.25% tween 20 with 4% methanol were used in control experiments. The effects of 8-methoxynaphthalen-1-ol on *S. lycopersicum* leaves from the leaf disk are shown in Table 1. Symptoms caused by compounds toward *S. lycopersicum* leaf disks at different concentrations after 18 h are shown in Fig. S5 while leaf absorption assay is shown in Fig. 4. From the results, 8-methoxynaphthalen-1-ol and the negative controls showed no visual effects on the leaves, while the positive control glyphosate exhibited phytotoxic effects on *S. lycopersicum* leaves at all concentrations.

**Table 1:** Phytotoxicity of compounds toward *S*. *lycopersicum* leaf disks at different concentrations after 18 h. A ‘0’ indicates no effect, ‘1’ indicates that area of necrotic spots <50%, ‘2’ is area of necrotic spots 50–70%, ‘3’ indicates area of necrotic spots 70–90%, and ‘4’ indicates that area of necrotic spots >90%.

Compound/herbicide	Concentration (µg/mL)			
	1,000	500	250	125
8-methoxynaphthalen-1-ol	0	0	0	0
Glyphosate	4	2	1	1
Distilled water	0			
Mixture of 0.25% tween 20 with 4% methanol	0			

### Phylogenetic analyses

**Figure 5:**
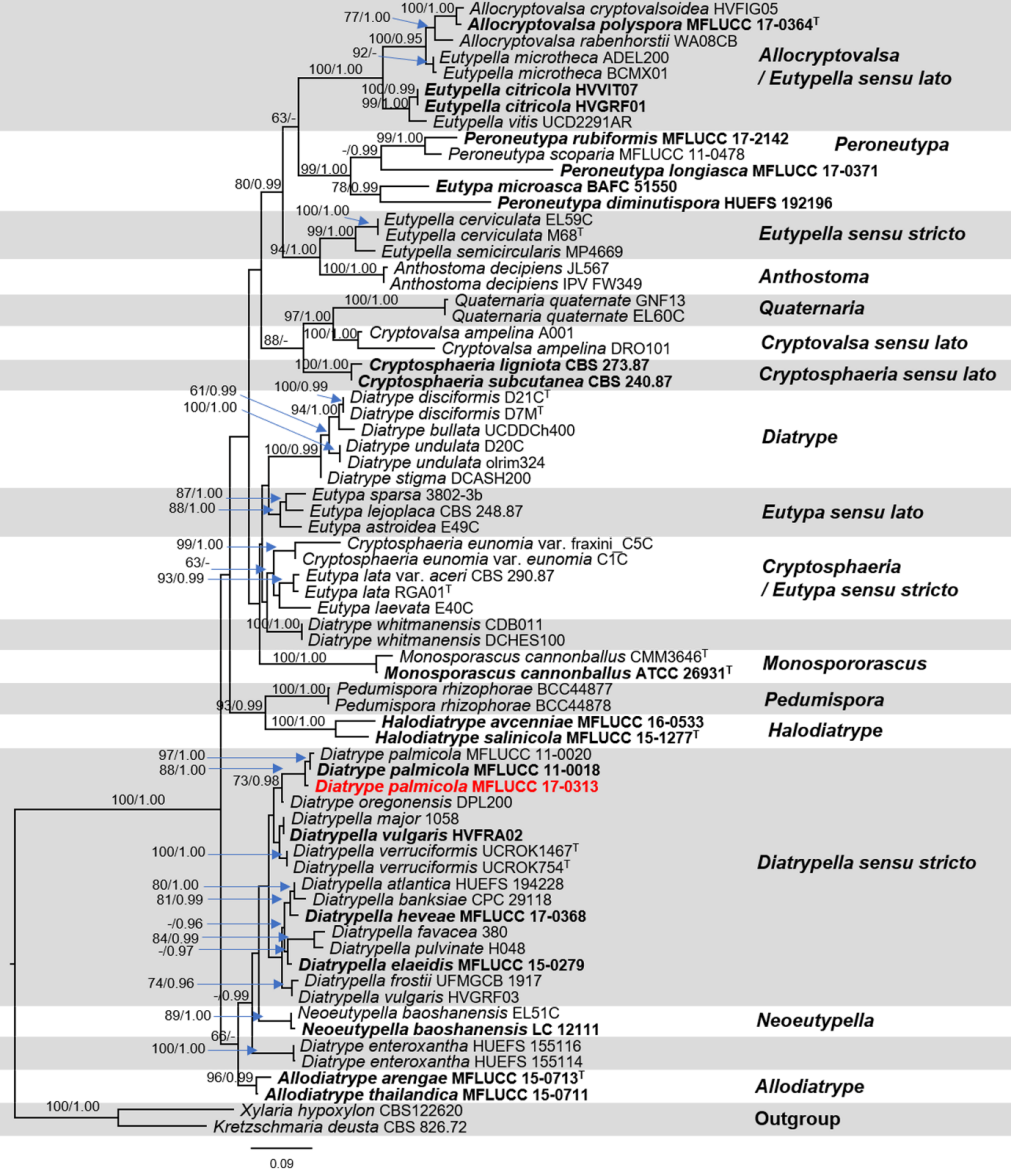
Phylogram generated from RAxML analysis based on a combined ITS and β-tubulin sequence data. Bootstrap support values for ML equal to or greater than 70%, and Bayesian posterior probabilities (PP) equal to or greater than 0.95 are defined as ML/PP above the nodes. The tree is rooted to *Xylaria hypoxylon* (CBS 122620) and *Kretzschmaria deusta* (CBS 826.72). All sequences from ex-type strains are in bold and type species are denoted with the superscript “T” after the species name. Newly generated strains are indicated in red.

The dataset consisted of 68 taxa including our strain and the outgroup taxa. RAxML analysis of the combined ITS and β-tubulin dataset had 1,246 distinct alignment patterns and 64.54% of undetermined characters or gaps. The best scoring of RAxML analysis shown in Fig. 5, with the final ML optimization likelihood value of −17386.379663. Bayesian posterior probabilities (BY) from MCMC were evaluated with the final average standard deviation of split frequencies = 0.010983. Phylogenetic analysis from ML, and BI gave trees with similar overall topologies of the generic placement and in agreement with previous studies (Senwanna et al., 2017; Konta et al., 2020). In the phylogenetic tree (Fig. 5), our taxon was clustered with *Diatrype palmicola* (MFLUCC 11-0018 and MFLUCC 11-0020) with 90% ML and 1.00 BY bootstrap support.

## Discussion

This study presented a similar result to those found in previous studies, which revealed that the endophytic fungi capable of producing bioactive compounds could be isolated from a wide range of plants (Shen et al., 2012; Golinska et al., 2015a; Golinska et al., 2015b). According to the results, the endophytic fungus MFLUCC 17-0313 displayed significant antifungal activity against *A. rolfsii*. The BLASTn search of ITS sequence shows more than 90% similarity with Diatrypaceae taxa (i.e., *Diatrypella banksiae* (CPC 29118), *Diatrypella atlantica* (HUEFS 194228), *Eutypella parasitica* (CBS 210.39) and *Diatrypella vulgaris* (CBS 128327)). In our pre-analyses of ITS sequence dataset, our strain was grouped within Diatrypaceae, however, only the ITS sequence is not appropriate to distinguish member in Diatrypaceae. Phylogram generated from RAxML analysis based on ITS sequence data (pre-analyses) is depicted in supplement information.

We, therefore, use the combined ITS and β-tubulin sequences in our dataset to clarify phylogenetic affinities of the genera in this family. As our attempts to obtain β-tubulin for our strain was unsuccessful. Therefore, here we do not include our β-tubulin sequence in analyses. Phylogenetic analyses of a combined ITS and β-tubulin sequence dataset revealed that the endophytic fungus MFLUCC 17-0313 grouped with *Diatrype palmicola* (MFLUCC 11-0018 and MFLUCC 11-0020) with moderate support (88% ML and 1.00 BYPP). Similar results with regards to support among Diatrypaceae members were obtained from previous studies (Acero et al., 2004; De Almeida, Gusmão & Miller, 2016; Shang et al., 2017; Senwanna et al., 2017; Konta et al., 2020). A comparison of ITS nucleotide bases showed that the endophytic fungus MFLUCC 17-0313 differed from *Diatrype palmicola* (MFLUCC 11-0018, type species) in 13/482 bp (2.7%). However, we could not obtain the reproductive structure in culture to compare the morphological characters of our strain with the type species. Despite this, for the time being our isolate will be treated as *Diatrype cf. palmicola* on the basis of the phylogenetic analysis. This taxonomic assignment is in agreement with Williams et al. (2008), who reported that fungi of the genus *Diatrype* produce secondary metabolites including lunalides A and B while treated with the DNA methyltransferase inhibitor 5-azacytidine. Both compounds were reported to possess substantial nematocidal activities (Cichewicz, 2012). In addition, *Diatrypella frostii* endophytic fungus of Diatrypaceae family has been reported to have antimicrobial activity such as isolated from Brazilian medicinal plant *Solanum cernuum* Vell (Vieira et al., 2011). Therefore, the Diatrype fungi isolated from medicinal plants could produce antifungal compounds.

The active compound, 8-methoxynaphthalen-1-ol, isolated from the crude hexane extract of the endophytic fungus MFLUCC 17-0313 mycelium was shown to have antifungal activity against *A. rolfsii*. This compound was reported in several studies. For instance, Rukachaisirikul et al. (2007) isolated this compound from the xylariaceous fungus PSU-A80 and found that it has good radical scavenging properties. Moreover, isomers and dimers of this compound were produced by several endophytes including the endophytic fungus *Nodulisporium* sp. from *Juniperus cedre* (Dai et al., 2006), endophytic fungal strain PR35 from *Paeonia delavayi* (Hu et al., 2012), and endophytic fungus *Daldinia cf. concentrica* (Liarzi et al., 2016). The literature reported the use of 8-methoxynaphthalen-1-ol as an antioxidative agent but no data have been published regarding the fungicidal activity of 8-methoxynaphthalen-1-ol.

In this study, the phytotoxic effect of 8-methoxynaphthalen-1-ol was effectively determined to evaluate its potential as biocontrol agent. The similar pattern of phytotoxicity of 8-methoxynaphthalen-1-ol was observed for all concentrations when the leaf disk and absorption assays were conducted. Toxicity data obtained from phytotoxicity tests are important for ecological risk assessment. These phytotoxicity tests are useful to inform regulators on the inherent toxicity of a given active compound. However, for 8-methoxynaphthalen-1-ol, its effects on species diversity, biodiversity, ecosystem services, and functions may be assessed in further studies.

## Conclusions

In summary, 34 endophytic fungal isolates were obtained from *P. fruticosa* leaves which is the first report of endophyic fungi from this plant. The antifungal activity against *A. rolfsii* of the endophytic isolates was investigated, leading to the isolation of the antifungal compound, 8-methoxynaphthalen-1-ol, from the endophytic fungus MFLUCC 17-0313 identified as *Diatrype palmicola*. The results showed that 8-methoxynaphthalen-1-ol was able to inhibit the growth of *A. rolfsii* and had an MIC value of 250 µg/mL. This bioactive compound possessed no phytotoxicity against *S. lycopersicum* and could be used as an alternative candidate for controlling *A. rolfsii* infection in tomatoes. The use of biofungicides will lead to a reduction in the amount of synthetic fungicides applied in agriculture.

##  Supplemental Information

10.7717/peerj.9103/supp-1 Supplemental Information 1 More figures and tables on antifungal activities, phytotoxicity test and raw data of active compound Fig. S1 Endophytic fungi were isolated from *P. fruticosa* leaves. All endophytic fungi were cultured in PDA at room temperature for two weeks. Fig S2 Antifungal activity of some endophytic fungi against *A. rolfsii* for seven days using dual culture assay for one week. Fig. S3 Antifungal activity of crude hexane extract (EXT), amphotericin B (drug), hexane (Hex) and control against *A. rolfsii* for seven days using disk diffusion assay. Fig. S4 Antifungal activity of some fractions, amphotericin B (drug), and control against A. rolfsii for seven days using disk diffusion assay. Fig. S5 Antifungal activity of pure compounds at different concentrations and amphotericin B (drug) against *A. rolfsii* for seven days using disk diffusion assay. Fig. S6 Symptoms caused by compounds toward *S. lycopersicum* leaf disks at different concentrations after 18 h. Table S1 Inhibition percentage of the all endophytic fungi against *A. rolfsii* from dual culture assay.

## Additional Information and Declarations

### Data Availability

The following information was supplied regarding data availability: The Diatrype sp. MFLUCC 17-0313 sequences are available at GenBank: MK329053.
